# Impact of Baseline Assessment Modality on Enrollment and Retention in a Facebook Smoking Cessation Study

**DOI:** 10.2196/jmir.4341

**Published:** 2015-07-16

**Authors:** Andrea C Villanti, Megan A Jacobs, Grace Zawistowski, Jody Brookover, Cassandra A Stanton, Amanda L Graham

**Affiliations:** ^1^The Schroeder Institute for Tobacco Research and Policy StudiesLegacyWashington, DCUnited States; ^2^Department of Health, Behavior and SocietyJohns Hopkins Bloomberg School of Public HealthBaltimore, MDUnited States; ^3^Milken Institute School of Public HealthThe George Washington UniversityWashington, DCUnited States; ^4^Department of OncologyGeorgetown University Medical Center/Cancer Prevention and Control ProgramLombardi Comprehensive Cancer CenterWashington, DCUnited States; ^5^WestatRockville, MDUnited States

**Keywords:** research subject recruitment, smoking cessation, Internet, social networking, adult

## Abstract

**Background:**

Few studies have addressed enrollment and retention methods in online smoking cessation interventions. Fully automated Web-based trials can yield large numbers of participants rapidly but suffer from high rates of attrition. Personal contact with participants can increase recruitment of smokers into cessation trials and improve participant retention.

**Objective:**

To compare the impact of Web-based (WEB) and phone (PH) baseline assessments on enrollment and retention metrics in the context of a Facebook smoking cessation study.

**Methods:**

Participants were recruited via Facebook and Google ads which were randomly displayed to adult smokers in the United States over 27 days from August to September 2013. On each platform, two identical ads were randomly displayed to users who fit the advertising parameters. Clicking on one of the ads resulted in randomization to WEB, and clicking on the other ad resulted in randomization to PH. Following online eligibility screening and informed consent, participants in the WEB arm completed the baseline survey online whereas PH participants completed the baseline survey by phone with a research assistant. All participants were contacted at 30 days to complete a follow-up survey that assessed use of the cessation intervention and smoking outcomes. Participants were paid $15 for follow-up survey completion.

**Results:**

A total of 4445 people clicked on the WEB ad and 4001 clicked on the PH ad: 12.04% (n=535) of WEB participants and 8.30% (n=332) of PH participants accepted the online study invitation (*P*<.001). Among the 726 participants who completed online eligibility screening, an equivalent proportion in both arms was eligible and an equivalent proportion of the eligible participants in both arms provided informed consent. There was significant drop-off between consent and completion of the baseline survey in the PH arm, resulting in enrollment rates of 32.7% (35/107) for the PH arm and 67.9% (114/168) for the WEB arm (*P*<.001). The overall enrollment rate among everyone who clicked on a study ad was 2%. There were no between group differences in the proportion that installed the Facebook app (66/114, 57.9% WEB vs 17/35, 49% PH) or that completed the 30-day follow-up survey (49/114, 43.0% WEB vs 16/35, 46% PH). A total of $6074 was spent on ads, generating 3,834,289 impressions and resulting in 8446 clicks (average cost $0.72 per click). Per participant enrollment costs for advertising alone were $27 WEB and $87 PH.

**Conclusions:**

A more intensive phone baseline assessment protocol yielded a lower rate of enrollment, equivalent follow-up rates, and higher enrollment costs compared to a Web-based assessment protocol. Future research should focus on honing mixed-mode assessment protocols to further optimize enrollment and retention.

## Introduction

Systematic reviews of recruitment methods for health studies [1-4] have highlighted online recruitment as a way to address many central enrollment challenges. Advantages of online recruitment include the ability to rapidly and cost-effectively reach a broad population, including those typically defined as hard to reach [5,6]. However, low retention (ie, loss to follow-up [7]) is of particular concern in fully automated, online trials where follow-up rates are often lower than in-person trials [8-10]. Internet-based trials have yielded an average follow-up rate of 53% for fully automated randomized trials, with some studies reporting rates as low as 13% [8].

Several approaches have been suggested to improve retention in Internet-based trials, including offline consent and data collection [10-13], modified online data collection formats [9,14-16], and filtering potential participants based on characteristics correlated with higher response rates [9,17-21]. There is evidence that online trials employing both online and offline follow-up methods may yield higher rates of follow-up [8,12,22,23].

This study used a randomized design to compare the effect of Internet and telephone baseline assessments on recruitment and retention metrics in a smoking cessation study involving Facebook. Our a priori hypothesis was that a more personalized assessment strategy at baseline (by phone) would depress enrollment rates and result in greater enrollment costs but would increase retention compared to a fully automated baseline assessment via the Internet. The study was conducted from August 2013 through November 2013 and was approved by the Schulman Associates Institutional Review Board.

## Methods

### Participants

Eligible participants had to be adult (18 years or older), self-identified smokers who were thinking of quitting in the next 30 days, had an active Facebook account, and had not used the smoking cessation Facebook app UbiQUITous [24].

### Recruitment, Enrollment, and Randomization

Facebook and Google AdWords advertisements were implemented simultaneously for 27 days from August 28 through September 24, 2013. All ads targeted adult smokers within the United States and were designed to be as similar as possible given the differences between Facebook and Google advertising platforms. On each platform, two identical ads were randomly displayed to users who fit the advertising parameters. Clicking on one of the ads resulted in randomization to the Internet baseline assessment (WEB), and clicking on the other ad resulted in randomization to the phone baseline assessment (PH).

A daily spending limit was set at USD $300 for all advertising. The bidding structure was set to maximize impressions in Facebook [25] and to maximize clicks in Google, with a max bid of $0.25/click. This advertising approach built on lessons learned by our research group from a previous randomized trial conducted within Facebook [24] and was consistent with each platform’s best practices at the time of the study [25-27].

Clicking on any of the ads took users to a study invitation page that provided a brief overview of the study and invited individuals to complete eligibility screening. Eligibility screening assessed gender, ethnicity, race, education, smoking status, motivation to quit, age, whether they had an active Facebook account, and whether they had used the Facebook app involved in this study. Only the last 5 questions determined eligibility; the other information was used to examine the characteristics of individuals responding to the recruitment ads.

Eligible participants completed online informed consent and provided contact information. Participants randomized to the WEB condition were immediately directed to the online baseline assessment; participants in the PH condition were informed that they would be contacted within 48 hours by a research assistant to complete the baseline assessment.

### Intervention

Following completion of the baseline assessment, all participants received information on how to install the study intervention, UbiQUITous, a Facebook app grounded in evidence-based smoking cessation treatment guidelines [28]. The development and implementation of the app is described in detail elsewhere [24].

### Assessment Procedures

The baseline survey comprised 14 questions addressing tobacco use, cessation-related cognitions and behaviors, and intensity of Facebook use [29]. It was deliberately short so as not to represent a barrier to study enrollment. At 30 days post-enrollment, participants in both arms were asked to complete a brief follow-up survey. Survey invitations were delivered via email, Facebook message, or telephone, with telephone follow-up for nonresponders. The survey assessed point prevalence abstinence (30-day and 7-day), tobacco use among those not abstinent, cessation intentions and quit methods used, and satisfaction with the UbiQUITous app. Participants were paid $15 for completion of the follow-up survey (Amazon gift certificate delivered via email).

### Outcomes

The outcomes of interest were the proportion enrolling in the study in the WEB compared to the PH arm and of those enrolled, the proportion completing the follow-up survey at 30 days (retention) in the WEB compared to the PH condition. Secondary outcomes were recruitment volume and enrollment costs.

### Statistical Analysis

This study used two sources of data: advertising metrics and enrollment and follow-up data extracted from our clinical trials management system. First, we developed a CONSORT diagram to track participants from ad exposure through enrollment and retention and estimated differences in proportions at each stage using chi-square tests. Second, we estimated the advertising cost per randomized participant in the WEB and PH conditions.

## Results

### Enrollment Metrics

Table 1 presents the flow of participants from online recruitment through follow-up. Overall, 4445 people were referred to the study from the WEB ad and 4001 from the PH ad; 12.04% (535/4445) of WEB participants and 8.30% (332/4001) of PH participants accepted the online study invitation (*P*<.001). There were no between-group differences in rates of eligibility or informed consent. The main reason for ineligibility was lack of intention to quit within the next 30 days (125 WEB, 57 PH). There was significant drop-off between consent and completion of the baseline survey in the PH arm, resulting in an enrollment rate of 32.7% (35/107) of PH participants versus 67.9% (114/168) of WEB participants (*P*<.001). The overall enrollment rate among everyone who clicked on a study ad was 1.76% (149/8446). Of the 149 participants who were enrolled, only 83 (55.7%) installed the Facebook app, with no differences between study arms (WEB: 66/114, 57.9% vs PH: 17/35, 49%; *P*=.33).

### Retention Metrics

As shown in Table 1, 43.6% of enrolled participants completed the 30-day follow-up survey, with no differences between study arms (WEB: 49/114, 43.0% vs PH: 16/35, 46%; *P*=.78).

### Enrollment Costs

During the recruitment period, a total of $6074 (WEB $3027; PH $3047) was spent on online recruitment ads, generating 3,834,289 (WEB 2,030,253; PH 1,804,036) impressions and resulting in 8446 (WEB 4445; PH 4001) participants clicking on the ads. Advertising costs per randomized participant were $27 WEB and $87 PH.

**Table 1:** Enrollment flow of participants.

Enrollment Step	Total, n (%)	WEB Arm, n (%)	PH Arm, n (%)	*P* value
Clicked on recruitment ad	8446 (100%)	4445 (100%)	4001 (100%)	—
Accepted study invite	867/8446 (10.3%)	535/4445 (12.0%)	332/4001 (8.3%)	<.001
Completed eligibility screening	726/867 (83.7%)	461/535 (86.2%)	265/332 (79.8%)	.014
Eligible	474/726 (65.3%)	292/461 (63.3%)	182/265 (68.7%)	.15
Consented	275/474 (58.0%)	168/292 (57.5%)	107/182 (58.8%)	.79
Enrolled	149/275 (54.2%)	114/168 (67.9%)	35/107 (32.7%)	<.001
Installed Facebook app	83/149 (55.7%)	66/114 (57.9%)	17/35 (48.6%)	.33
Completed follow-up	65/149 (43.6%)	49/114 (43.0%)	16/35 (45.7%)	.78

## Discussion

### Principal Findings

This study used a novel randomized design to assess the impact of baseline assessment method on recruitment and retention. Our main finding was that a more intensive phone baseline assessment protocol yielded a lower rate of enrollment, equivalent follow-up rates, and higher enrollment costs compared to a web-based baseline assessment protocol. The overall enrollment rate of 2% was smaller than other trials but still in the range of 6% observed in another large web-based randomized trial [13]. Overall retention at 30 days in our study was 44%, similar to previous literature on web-based randomized trials [8] and cohort studies [30] but lower than reported in other web-based cessation studies with longer follow-up periods [13,31]. The equivalent retention rates in the web and phone arms are consistent with findings reported in a weight management study [32]. Our enrollment costs were also in line with recent studies recruiting via Facebook ads [26,27].

### Strengths and Limitations

While our study suffered from low enrollment, differential drop-out between study arms, and low retention, these metrics were in line with those reported in other online trials [8,13]. Of interest to others conducting online recruitment, we found Google AdWords to be an ineffective recruitment strategy driven by automated shut-off by Google due to poor performance. Possible barriers to performance from the Google ads may have been the frame shift needed from an Internet search query to Facebook app installation (versus Facebook ad for a Facebook app) or the design of the ads, which attempted to keep content and pricing similar in order to directly compare the yield of Facebook to Google advertising. Researchers using online ads across different platforms for study recruitment should take these concerns into consideration when designing and implementing recruitment protocols.

One strength of our study is the randomization of participants to recruitment method using the underlying auction mechanism to place ads within Facebook and Google. Our study is one of the first to explicitly examine the impact of enrollment strategies on recruitment and retention metrics within the context of online intervention research. Future research should focus on honing advertising strategies and web-based assessment protocols to further optimize enrollment and retention.
